# Kagami–Ogata syndrome in a fetus presenting with polyhydramnios, malformations, and preterm delivery: a case report

**DOI:** 10.1186/s13256-019-2298-y

**Published:** 2019-11-22

**Authors:** Haipeng Huang, Yukiko Mikami, Kosuke Shigematsu, Nozomi Uemura, Mamiko Shinsaka, Ayaka Iwatani, Fumihito Miyake, Kazuhiko Kabe, Yasushi Takai, Masahiro Saitoh, Kazunori Baba, Hiroyuki Seki

**Affiliations:** 1 Department of Obstetrics and Gynecology, Saitama Medical Center, Saitama Medical University, 1981 Kamoda, Kawagoe City, Saitama 350-3550 Japan; 2 Department of Pediatrics, Saitama Medical Center, Saitama Medical University, Kawagoe, Japan

**Keywords:** Kagami–Ogata syndrome, Genetic diagnosis, Epigenetic mutation, Amniodrainage, Fetal diagnosis

## Abstract

**Background:**

Kagami–Ogata syndrome is also known as paternal uniparental disomy 14 and related disorders and is caused by abnormal genomic imprinting in the long arm of the chromosome 14q32.2 region. Its clinical manifestations include polyhydramnios in the fetal stage, respiratory insufficiency because of a small thorax, abdominal wall abnormalities, and peculiar facial features after birth.

**Case presentation:**

A 38-year-old Japanese primigravida woman was referred to our hospital in the 19th week of pregnancy for suspected omphalocele. She had a history of hypothyroidism but was prescribed orally administered levothyroxine (50 μg/day) prior to conception and was euthyroid. Her ultrasound scan prior to visiting our hospital revealed fetal omphalocele, heavy for date, and polyhydramnios. The mother was advised to be admitted for observation from 28 weeks of gestation for threatened premature delivery. She required amniodrainage at 29 and 32 weeks of gestation. At 35 weeks of gestation, the fetal membrane prematurely ruptured and she gave birth after an emergency Cesarean section. The infant was a male child with a birth weight of 3188 g, and was suspected to have Kagami–Ogata syndrome after birth based on thoracic hypoplasia, swallowing function abnormalities, and peculiar facial features. A definitive diagnosis was established by performing genetic testing of the infant after obtaining informed written consent from both the parents; the results of the genetic testing revealed hypermethylated intergenic-differentially methylated region and maternally expressed gene 3-differentially methylated region in the corresponding chromosome 14 region. Both the parents were genetically tested after adequate genetic counseling, which revealed a *de novo* microdeletion in a differentially methylated region.

**Conclusion:**

Kagami–Ogata syndrome should have been suspected because of the presence of polyhydramnios and omphalocele during pregnancy. Respiratory insufficiency soon after birth, because of a small thorax, is expected in this disease and a diagnosis during pregnancy may have enabled appropriate care after birth.

## Background

Kagami–Ogata syndrome is caused by abnormal genomic imprinting in the long arm of the chromosome 14q32.2 region. Genes are normally passed on to the offspring equally from both the parents; however, in genomic imprinting, genes are inherited from either the father (paternally expressed gene) or mother (maternally expressed gene) [1]. The imprinted region of chromosome 14 has two differentially methylated regions (DMRs) that function as imprinting centers. These two intergenic (IG)-DMRs are established at the time of gametogenesis, whereas maternally expressed gene 3 (*MEG3*)-DMR is established after fertilization. Normally, these regions are hypermethylated in the case of paternally expressed genes and hypomethylated in the case of maternally expressed genes; as a result, *RTL1* expression is suppressed in the maternal disomy of chromosome 14. However, in the paternal disomy of chromosome 14, two alleles of DMR are hypermethylated, resulting in overexpression of *RTL1*, which is responsible for clinical phenotypes. Other known causes apart from disomy are epigenetic mutations and microdeletions, including DMR of maternal origin [2, 3].

The clinical phenotypes of cases have been reported previously. Specific facial gestalt (full cheeks and protruding philtrum, forehead protrusion, blepharophimosis, flat nasal bridge, micrognathia, forward-facing nostrils), thoracic hypoplasia (higher coat hanger angle in a chest X-ray, bell-shaped chest), abdominal wall abnormalities (omphalocele, diastasis recti, and so on), polyhydramnios, placental hyperplasia, overweight at birth, psychomotor retardation, and feeding dysphagia, among other phenotypes, have been reported [2]. The diagnosis of the present case was confirmed based on the above-mentioned clinical phenotypes and using a genetic diagnosis flowchart.

Although this was a very rare case, Kagami–Ogata syndrome can reportedly be diagnosed at the fetal stage. We, retrospectively, investigated this case to evaluate the clues for fetal diagnosis of this syndrome.

## Case presentation

Our patient was a 38-year-old Japanese primigravida woman referred to this hospital in the 19th week of pregnancy for suspected omphalocele. Her blood pressure was normal, height was 169 cm, body weight was 58 kg, body mass index (BMI) was 22, and she had no lower limb edema. She had a history of hypothyroidism but was prescribed orally administered levothyroxine (50 μg/day) prior to conception and was euthyroid. An ultrasound (US) scan prior to her referral to this hospital revealed fetal omphalocele. The estimated fetal weight was 365 g (+ 2.0 SD), which was large for date, and amniotic fluid was 130 cm, indicating polyhydramnios. Middle cerebral artery (MCA) and umbilical artery (UA) Doppler studies revealed an MCA-resistance index (RI) of 0.89, MCA-peak systolic velocity (PSV) of 49.07 cm/second, and UA-impedance index (II) of 0.49. The 75 g glucose tolerance test was conducted and the result was found to be normal. The mother was advised to be admitted for observation from 28 weeks of gestation for threatened premature delivery. She had a feeling of abdominal tightness but had no breathing difficulty; the cervical length was 15 mm. She was given betamethasone (12 mg per 24 hours, twice) because of the likelihood of preterm delivery at 31 weeks of gestation because of difficulty in controlling uterine contractions. In addition, because of aggravation of pressure symptoms due to polyhydramnios, we performed transabdominal amniocentesis and amniodrainage at 29.4 and 32.4 weeks without complications and removed 1535 mL and 1126 mL of amniotic fluid, respectively. She suffered premature rupture of the fetal membrane at 35.1 weeks and gave birth on the same day by emergency Cesarean section.

**Fig. 1:**
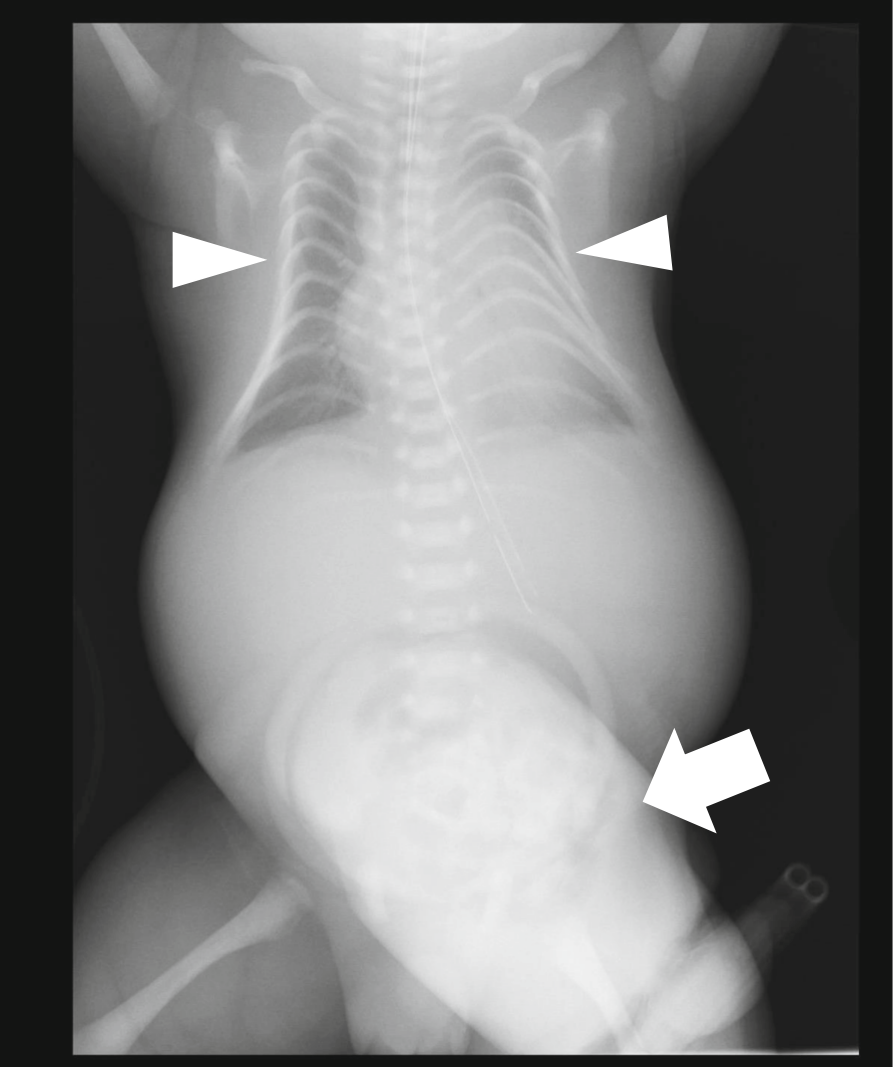
Plain chest radiograph taken 3 hours after birth showing the thoracoabdominal frontal view. The thorax is small and bell-shaped (*arrowheads*) and the ribs are deformed with the same shape. The abdomen shows omphalocele and intestinal gas can be observed inside the hernial sac (*arrow*)

The infant was a male child with a birth weight of 3188 g, Apgar scores of 3 at 1 minute and 6 at 5 minutes, and UA pH of 7.383. Spontaneous respiration of the infant was poor, and we performed intratracheal intubation at 9 minutes after birth and started artificial ventilation for respiratory care. A chest X-ray taken at 3 hours after birth (Fig. 1) showed a bell-shaped chest, small in proportion with respect to the body, as well as deformed ribs. We also detected generalized hypotonia, a flat nasal bridge, and a small jaw, suggesting the presence of a chromosomal abnormality. The infant also had an abnormal-looking face.

Immediately following birth, we treated the omphalocele by hygienically safeguarding the umbilical hernia section using a wound retractor folded into a tent shape to prevent damage to the hernial sac, and then waited for the hernial contents (in this case, small intestines) to naturally reduce into the abdominal cavity. Thereafter, at 10 days of age, we performed a radical surgery for the omphalocele.

At 13 days of age, we performed endotracheal extubation and the infant was moved to bi-level positive airway pressure. Thereafter, he gradually stopped requiring positive pressure, and spontaneous respiration was established using nasal oxygen administration alone. Enteral feeding was started at 12 days of age. Thereafter, upon trying to feed milk orally, swallowing dysfunction was detected. Enteral feeding (full feeding) was established at 20 days of age along with commencement of swallowing training.

Because the infant showed omphalocele, polyhydramnios, overweight, and abnormalities identified after birth, that is, thoracic hypoplasia morphological abnormality of the ribs, abnormal swallowing function, and peculiar facial features, we suspected Kagami–Ogata syndrome. After obtaining informed written consent from both the parents, chromosomal banding and genetic testing was performed for the infant.

Chromosome G-banding showed 46 normal XY karyotypes. Genetic testing revealed hypermethylated IG-DMR and *MEG3*-DMR in the corresponding chromosome 14 imprinted regions. He was, therefore, diagnosed as having Kagami–Ogata syndrome. Furthermore, genetic testing of both the parents was performed after adequate explanation and genetic counseling. Although the alleles in the infant patient originated from both the parents, a comparative genomic hybridization (CGH) array showed microdeletion in the imprinted region. Analyses of both the parents revealed no deletion; thus, the deletion in the infant patient was considered *de novo*.

The infant was discharged at 90 days of age with domiciliary oxygen therapy. After leaving the hospital, he used home oxygen therapy until he was 1 year and 3 months old, and after that, his breathing condition calmed down. He was able to walk alone at the age of 1 year and 8 months. A delay in language development was noticed and at the age of 2 years and 5 months, no meaningful speech could be recognized.

## Discussion and conclusions

Literature on fetal diagnosis reveals that seven or eight cases of death occur in premature infants [4]. Polyhydramnios, which is frequently associated with this syndrome, increases the risk of premature birth; therefore, prolonging the gestation during perinatal period management may reduce the incidence of infant death. Moreover, severe postnatal respiratory insufficiency occurs because of thoracic hypoplasia but tends to improve as the chest matures; therefore, it is essential to identify the disease during the fetal stage so that appropriate and adequate care can be provided after birth. Although Kagami–Ogata syndrome is rare, a previously reported case was diagnosed in the fetal stage. According to this report, the thoracic disorder was identified by X-ray photography during the fetal stage, leading to a valid diagnosis [5].

**Fig. 2:**
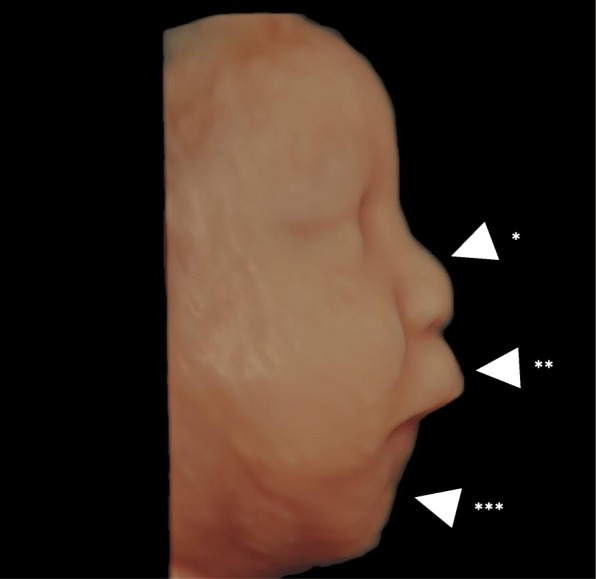
Ultrasound three-dimensional image of the face of the fetus taken at 31 weeks of gestation. The fetal face viewed from the right side shows a flat nose bridge (arrowhead*), protruding philtrum (**), and a small jaw (***)

**Fig. 3:**
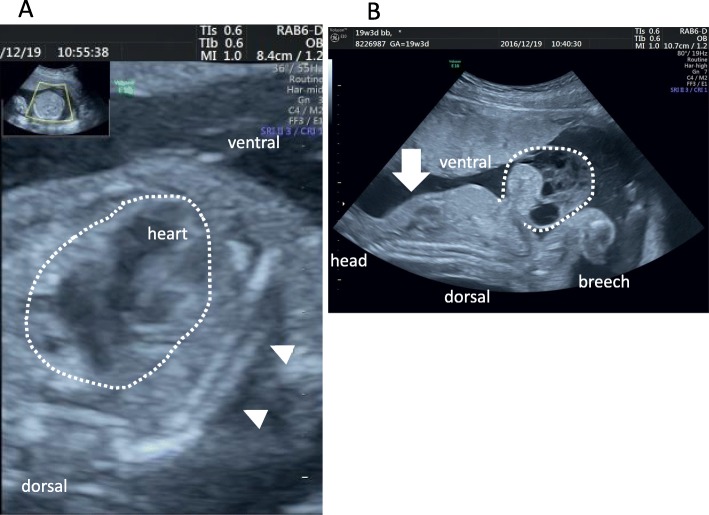
**a** B-mode ultrasound at 19 weeks of gestation showing transverse section of the chest. It shows straight-line ribs (*arrowhead*) and lungs (*arrow*) that are smaller in proportion to the heart (*dotted line*). **b** B-mode ultrasound at 19 weeks of gestation showing a sagittal section of the thoracoabdominal region. It shows omphalocele (*dotted line*) and digestive tract penetrating into the hernia. The chest is smaller than the abdomen, leading to suspicion of thoracic hypoplasia

In this case, we retrospectively examined the points at which fetal diagnosis could have been established. In this case, omphalocele, polyhydramnios, and overweight were observed in the fetal stage. Re-examination of the three-dimensional/four-dimensional ultrasonogram revealed that the forehead and nose shape were characteristic of Kagami–Ogata syndrome (Fig. 2). US examination also showed that the costal arch was in a straight line (Fig. 3), suggesting thoracic hypoplasia. Despite the omphalocele, the abdominal wall was raised more than the chest wall, which may have indicated the presence of the syndrome. If all these points had been comprehensively analyzed, the diagnosis could have been reached during the pregnancy.

In conclusion, cases presenting with abnormalities of the abdominal wall, such as polyhydramnios and omphalocele, and abnormality of the costal arch must be medically examined to rule out Kagami–Ogata syndrome. If diagnosed during pregnancy, the neonatal department can be consulted, information can be provided to both the parents, and the infant can be provided with continuous medical treatment after birth. Prenatal diagnosis may also be helpful for proactive treatment for threatened premature delivery.

## Data Availability

Not applicable.

## Abbreviations

BMI: Body mass index
CGH: Comparative genomic hybridization
DMR: Differentially methylated region
IG: Intergenic
II: Impedance index
MCA: Middle cerebral artery
*MEG3*: Maternally expressed gene 3
PSV: Peak systolic velocity
RI: Resistance index
UA: Umbilical artery
US: Ultrasound
